# Computing Temporal Sequences Associated With Dynamic Patterns on the *C. elegans* Connectome

**DOI:** 10.3389/fnsys.2021.564124

**Published:** 2021-03-09

**Authors:** Vivek Kurien George, Francesca Puppo, Gabriel A. Silva

**Affiliations:** ^1^Department of Bioengineering, University of California, San Diego, San Diego, CA, United States; ^2^Center for Engineered Natural Intelligence, University of California, San Diego, San Diego, CA, United States; ^3^BioCircuits Institute, University of California, San Diego, San Diego, CA, United States; ^4^Department of Neurosciences, University of California, San Diego, San Diego, CA, United States

**Keywords:** connectome analysis, *C. elegans* model, computational modeling, graph theory, networks (circuits)

## Abstract

Understanding how the structural connectivity and spatial geometry of a network constrains the dynamics it is able to support is an active and open area of research. We simulated the plausible dynamics resulting from the known *C. elegans* connectome using a recent model and theoretical analysis that computes the dynamics of neurobiological networks by focusing on how local interactions among connected neurons give rise to the global dynamics in an emergent way. We studied the dynamics which resulted from stimulating a chemosensory neuron (ASEL) in a known feeding circuit, both in isolation and embedded in the full connectome. We show that contralateral motorneuron activations in ventral (VB) and dorsal (DB) classes of motorneurons emerged from the simulations, which are qualitatively similar to rhythmic motorneuron firing pattern associated with locomotion of the worm. One interpretation of these results is that there is an inherent—and we propose—purposeful structural wiring to the *C. elegans* connectome that has evolved to serve specific behavioral functions. To study network signaling pathways responsible for the dynamics we developed an analytic framework that constructs Temporal Sequences (TSeq), time-ordered walks of signals on graphs. We found that only 5% of TSeq are preserved between the isolated feeding network relative to its embedded counterpart. The remaining 95% of signaling pathways computed in the isolated network are not present in the embedded network. This suggests a cautionary note for computational studies of isolated neurobiological circuits and networks.

## 1. Introduction

A variety of network analyses methods are widely used to study complex systems (Newman, 2010; Varshney et al., 2011; Deo, 1975; Bassett et al., 2017; Boccaletti et al., 2014, 2006; Sporns et al., 2005). But understanding how the structural connectivity of a network constrains the dynamics it is able to support is still an active and open area of research. This is particularly the case for spatial-temporal networks (Holme, 2015), where much of the literature and methods are still in early stages and mostly descriptive, providing few tools for predictive modeling. As a result, the application of this emerging body of work toward the treatment and analyses of biological neural networks that are inherently spatial-temporal remains quite limited. But the theoretical and practical insights that such an approach would offer neurobiology are potentially very significant. One such application is the analyses of the dynome (Kopell et al., 2014), that is, the combination of the structural connectome and dynamic model. In this work, we introduce a novel set of methods and analyses for this task, and apply it to study the dynamics of the *Caenorhabditis elegans* worm connectome as an example. This remains at the forefront of neuroscience research (Bargmann and Marder, 2013; Buonomano and Maass, 2009).

The *C. elegans* connectome consists of 302 neurons and their anatomical links (White et al., 1986; Hall and Altun, 2008). Although the organism's connectome has been known for decades, how neurons functionally interact in the context of the entire network and how the resultant dynamics regulates participating neurons is not fully understood (Bargmann, 2012; Bargmann and Marder, 2013; Larson et al., 2018). A recent tend in *C. elegans* research is to study the dynome (Kopell et al., 2014; DiLoreto et al., 2019; Towlson et al., 2018), rather than drawing inferences from just the structural connectivity of the network (Towlson et al., 2013; Varshney et al., 2011; Sabrin et al., 2019).

A considerable body of *C. elegans* research focuses on the functional consequences of the dynome (Sabrin et al., 2019; Kato et al., 2015; Brennan and Proekt, 2019; Moreira and de Aguiar, 2019; Szigeti et al., 2014; Yan et al., 2017; Kim et al., 2019; Sarma et al., 2018). Experimentally and computationally researchers have performed subnetwork and whole worm analysis (Kato et al., 2015; Kim et al., 2019; Kaplan et al., 2018; Kunert-Graf et al., 2017). Along the vain of current research, we propose methods to answer the following question: How does concurrent activity of independent neuronal elements (nodes), along axons (edges) ultimately give rise to a rich behavioral repertoire? Our methods are focused on an understudied topic of *C. elegans* research, that is, the dynamic implications of the spatial separation of neurons. This is stymied by experimental findings which indicate that many *C. elegans* neurons do not communicate using traditional action potentials (Lockery and Goodman, 2009; Goodman et al., 1998; Kandel et al., 2013), with few exceptions (Mellem et al., 2008; Shindou et al., 2019; Liu Q. et al., 2018). Several works dedicated to studying the *C. elegans* dynome use biophysically plausible node models which seek to emulate experimental voltage responses (Kunert et al., 2014; Nicoletti et al., 2019). Researchers performed whole worm analysis from a variety of perspectives including probabilistic graphical models (Liu H. et al., 2018), dynamical systems theory (Kunert-Graf et al., 2017), and state space analysis (Linderman et al., 2019). Rather than focusing on recreating the voltage dynamics on the connectome, we assumed that communication between neurons is constrained by the axonal conduction velocity, that coupled with the spatial separation of neurons, results in edge signal delays.

We modeled the signaling dynamics in the network as the passing some discrete quanta of charge, at finite speeds, between neurons, along directed edged. As such, node interactions are constrained by the network's connectivity, spatial geometry, and signaling parameters. This analysis is built upon a model and framework posited by a recently published paper (Silva, 2019) which focused on how local interactions among connected nodes give rise to global dynamics in an emergent way. This framework was derived from canonical principles of spatial and temporal summation in biological neurons. The network interactions are governed by the arrival times of incident signals into nodes and how those signals compete to activate downstream nodes. We used this methodology to compute the network dynamics on the *C. elegans* connectome.

We constructed a geometric network by combining the *C. elegans* wiring diagram–focusing on axonal connectivity–with node location data to calculate euclidean straight-line edge lengths (Varshney et al., 2011; White et al., 1986; Kaiser and Hilgetag, 2006; Choe et al., 2004). We used the edge lengths and signal conduction velocities (Niebur and Erdo, 1993) to ascertain the edge signal delays. This is the amount of time it takes for a signal to traverse between two connected neurons. Every node was endowed with the same refractory period within biological range. Finally, each node responded in either an excitatory or inhibitory manner. In section 4.1, we describe in detail how we constructed the Full network.

Using the aforementioned geometric network model, we studied the dynamics which results from stimulating the chemosensory neuron ASEL. This neuron is known to detect the presence of food (Pierce-Shimomura et al., 2001; Suzuki et al., 2008). Laboratory experiments have shown that the activation of ASEL ultimately results in a movement response (Pierce-Shimomura et al., 2001; Suzuki et al., 2008; Wang et al., 2017). *C. elegans* locomotion is produced by the synchronized activation of mid-body motor neurons (Haspel et al., 2010; Zhen and Samuel, 2015). We chose ASEL because it is both well-studied and because it belongs to a hypothesized subnetwork (Xu and Deng, 2013)–the Feed network–whose dynamics we were interested in interrogating on a standalone basis and relative to the Full network.

We found that a geometric embedding increases the dynamical repertoire of the network, as measured by the number of unique network states. We quantified number of unique network states between a spatially-aware network–one whose edge delays are derived from node location data–and a spatially-unaware network–one whose edge delays are all the same. Interestingly, stimulating ASEL led to dynamics on the spatially-aware network eventually resulted in contralateral motor neuron activation in the ventral (VB) and dorsal (DB) classes of motor neurons. This rhythmic alternating back-and-forth motor neuron firing pattern is qualitatively indicative of that required for movement of the worm (Haspel et al., 2010; Xu and Deng, 2013; Zhen and Samuel, 2015). This result is subtle but critical in its interpretation. It is important to realize that we did not in any way model or intentionally try to emulate such rhythmic oscillatory dynamics between these two motorneuron populations. This dynamic behavior was in response to a single impulse input into the network (via ASEL), and reflects the inherent (and we propose) purposeful structural wiring of the *C. elegans* connectome that has evolved over a very long time to serve purposeful behavioral functions. Furthermore, the Feed network also exhibited similar motorneuron response.

To uncover why the Feed and Full networks give rise to similar contralateral motor activation patterns, we identified and analyzed the signaling paths beginning at ASEL to the VB and DB neurons. This reflects a unique part of the work which was enabled by the way our dynamic framework and simulation model were constructed. Neuronal signaling paths encode the causal chain of node activations over the course of the dynamics. To describe each signaling path we introduced the notion of a Temporal Sequence (TSeq). Each TSeq is a temporally ordered sequence of nodes, formally a walk on a graph (Diestel, 2006). We developed a method to quantitatively compare TSeqs, which we used to determine the extent to which subnetworks (the Feed network) preserve causal signaling paths present in the Full network. Furthermore, we decomposed the complex network activity into a compact basis set of TSeqs. This set plays two roles, it can be used a signature of the network dynamics, and it can be used to construct subnetworks which more closely resemble the signaling dynamics of the larger network. Finally, upon analysis of the TSeqs responsible for VB and DB activity, we showed the prevalence of different classes of neurons from the motor circuit.

The focus of this paper is to show that a combination of a geometric network, signaling framework, and TSeqs—when applied to analyze the affects of stimulating ASEL—resulted in some surprising findings. In addition, the computational workflow we developed can be used to generate experimentally testable hypothesis regarding concurrent network activity. Moreover, TSeqs can be used to generate a set of candidate subnetworks for more computationally intensive and detailed studies. Outside neuroscience, we are using these methods to analyze the causal dynamics on complex networks for machine learning. For example, in image classification setting, we can encode images through causal network dynamics in the from of TSeqs. Thus, the methods we developed extend beyond computational neurobiology and are useful in any network context where causal interactions can be delineated.

## 2. Results

### 2.1. The Contribution of Network Geometry on Dynamics

We found that a geometric embedding of nodes in the network plays an important role in network dynamics (Silva, 2019; Buibas and Silva, 2010). A signal's finite conduction velocity and a network's physical geometry, i.e., convoluted paths in space, results in edge signal delays which adds significant richness to the resultant network dynamics. This is due to the offset in signal arrival times at nodes and the subsequent effects that has. Within the context of our model, we quantified the affect of signal delays on network dynamics in *C. elegans*.

**Figure 1:**
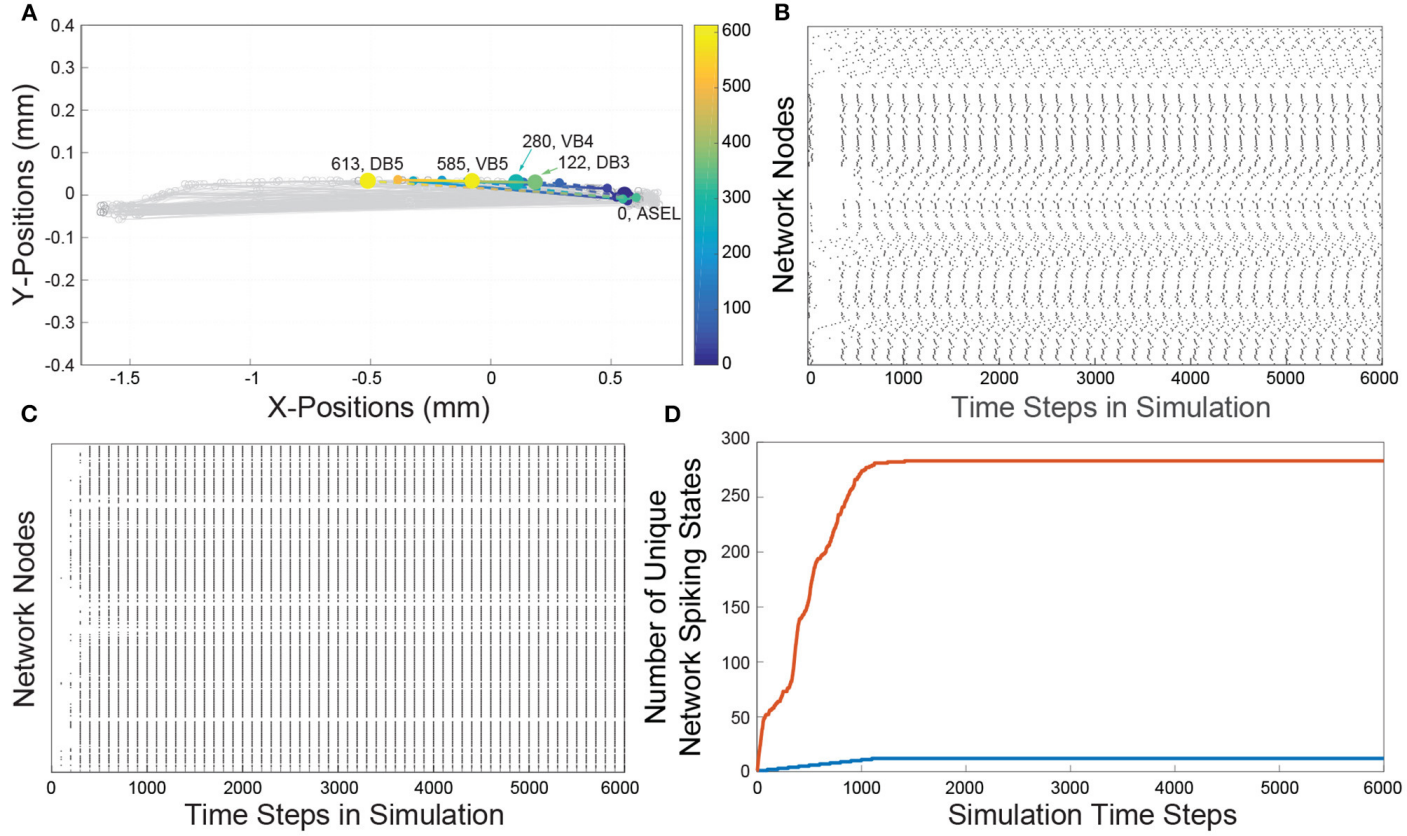
**(A)** A reconstruction of the *C. elegans* connectome. Each gray line represents an edge. Each edge represents chemical connections between nodes. The colored dashed lines represents a sample of the traversal of a signal from the sensory neuron ASEL through the interneurons in the network to the VB and DB classes of motorneurons. The color gradient indicates the time course as the signal traverses the network. Cold colors represent early in the neuronal signaling pathway and warm colors represent late in the neuronal signaling pathway. The node labels and numbers next to them represent the names of the motorneurons at the terminus of the sequence of activations and the time step when that terminal node was activated. **(B)** The Full network's spike raster resulting from ASEL stimulation. The activity is a direct result of the signaling parameters (signal conduction velocity, refractory period), network connectivity, and the spatial locations of each of the nodes. Each dot represents the activation of a node in the network, the white space represents an inactive state of the node. Each discrete height on y-axis represents a specific node in the network. **(C)** The Lattice network's spike raster caused by stimulating ASEL. The Lattice network preserves the signaling parameters and network connectivity, but has modified spatial node locations relative to the Full network. We set the delays for all edges in the Lattice network to be the same. The resulting spike raster is significantly less varied than that of the Full network. **(D)** Each curve is a cumulative count of the number of unique network states of presented by the Full (red curve) and Lattice (blue curve) networks as the dynamics proceeds over time. There are approximately a 20 times more network states in the dynamics of the Full network than dynamics of the Lattice network.

We compared the network activity of the spatially embedded *C. elegans* connectome (Figure 1A), henceforth referred to as the Full network (its construction is described in section 4.1), to the activity resulting from a *C. elegans* connectome whose nodes are arranged in a spatial lattice, this network is referred to as the Lattice network (its construction is described in section 4.3). While the Lattice network's edge connectivity and node types (inhibitory or excitatory) are identical to the Full network, we set all the Lattice network's edge signal delays to a constant value; this then eliminates the effects of spatial embedding on the dynamics.

In Figures 1B,C, we show the network activity of the Full and Lattice networks, that results from a single pulse stimulus at the ASEL neuron. This effectively represents the simplest stimulus and input into the network that is possible within the singling framework. We observed that the Lattice network's activity went through a transient period of node activity, but over time, all possibly activated nodes were firing at the same time, in a periodic manner (Figure 1B). In contrast, although the Full network's node activity also went through transient and periodic phases, we observed a greater number and variability of patterns in its network states (Figure 1C). To quantify the affect on network activity due to network geometry, we counted the number of unique network states exhibited by each network. We describe the network's state through a vector of nodal states at each simulation time point. Concretely, if the state of node *i* at time *t* is given by *y*_*i*_(*t*) = {0, 1}, then the state of a network, with *N* nodes, at time *t*, can be written as **y**(*t*) = {0, 1}^*N*^. In Figure 1D, we show the cumulative count of unique network states over the course of the simulation. The Lattice network assumed 12 unique states, while the Full network assumed 280 unique states across the sample time points (we did not consider permutations of consecutive states).

These results suggest that the geometry is an important consideration because of it increases dynamic range of the network. The spatial network embedding gave rise to seemingly coherent patterns of temporal network activity (Figure 1B). In what follows, we qualitatively and quantitatively study this network activity in the context of various networks.

### 2.2. Qualitative Comparison of Network Activity Between the Full and Feed Networks Using Temporal Sequences

The behavioral consequence of the activation of the ASEL neuron is movement (Xu and Deng, 2013; Suzuki et al., 2008; Wang et al., 2017). This is achieved by synchronized contralateral periodic firing in alternating populations of motorneurons (Haspel et al., 2010; Zhen and Samuel, 2015). To do comparisons of network activity, we used a previously delineated subnetwork (Xu and Deng, 2013) that contain nodes which transduces a food stimuli—through ASEL activation—to a movement response, which is indicated by the activity of the VB and DB populations of motorneurons. As such, to compare node activity across networks various networks, we focus on the neural activity of VB and DB motorneuron populations which are present in both the Full network and the Feed network. The construction of the Feed network detailed in section 4.2.

**Figure 2:**
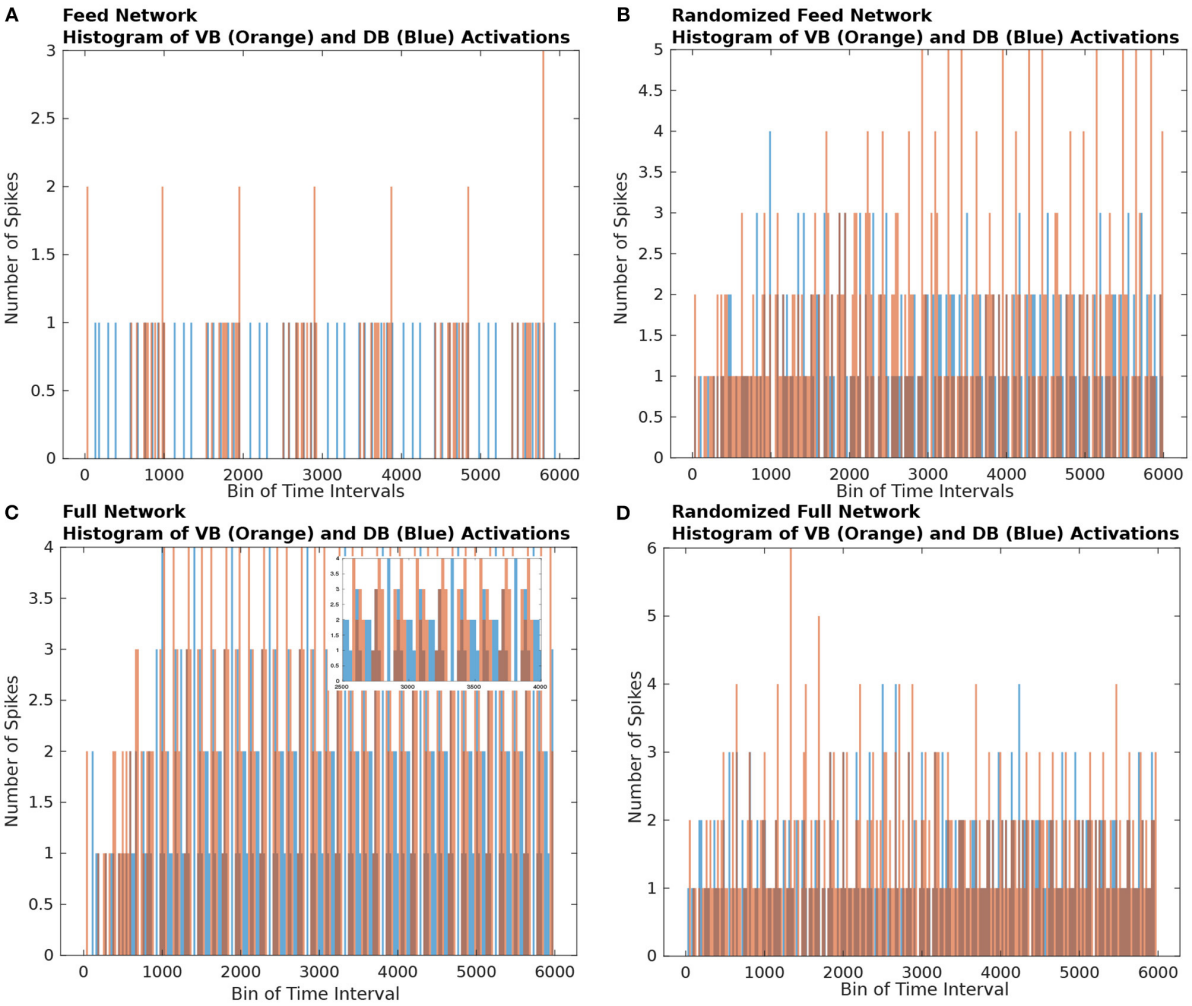
Histograms of the number of firings of nodes from the VB (orange rectangles) and DB (blue rectangles) classes. The bin size of the histogram is 250 time steps. **(A)** Histogram of the Feed network's VB and DB class motorneurons activations. We observe that the VB and DB classes of motorneurons activate in an alternating pattern. **(B)** Histogram of the Gilbert Randomized Feed network's VB and DB class neurons activations. Because the randomization procedure significantly modified the network connectivity, the class based alternating neuronal activation pattern was decimated. We observe a semblance of periodic node activations from the VB and DB classes after approximately 3000 time steps. **(C)** Histogram of the Full network's VB and DB class neurons activations. The *C. elegans* connectome gives rise to significantly more activity in the VB and DB classes of motorneurons in comparison to the activity in the Feed network **(A)**. The dynamics on the Full network also supported alternating VB, DB class activity. Because of the density of node activation, in the inset we show a zoomed in view of a sub-interval of activity to better view the VB-DB class alternating neuronal activation pattern. **(D)** Histogram of the Gilbert Randomized Full network's VB and DB class neurons activations. The randomization procedure resulted in the destruction of the class-wise alternating neuronal activation pattern.

In Figures 2A,C, we show the time-binned histogram of VB and DB motorneuron activity which results from stimulating ASEL in the Feed and Full networks, respectively. We observed that the VB and DB motorneurons activated in a staggered back and forth synchronized contralateral periodic manner. This activity pattern is qualitatively similar to the contralateral firing patterns necessary for the locomotion of the worm (Zhen and Samuel, 2015; Haspel et al., 2010; Riddle et al., 1997). Both the Full and the Feed networks preserved the general patterns of ventral and dorsal neuronal activity.

To qualitatively show that the network's activity is affected by edge connectivity, we constructed randomized networks—called the Gilbert Randomized networks—derived from the Feed and Full networks, then simulated dynamics on each of them. The Gilbert Randomized networks were constructed using a randomization procedure (Gilbert, 1959; Erdos and Renyi, 1960) which preserved each node's spatial location, but replaced the original set of edges with a new set, which is a randomly drawn subset of the allowed edges between nodes (a detailed description of its network construction can be found in section 4.5). Despite the edge randomization, stimulating the ASEL in the Gilbert Randomized networks still led to VB and DB population activity (Figures 2B,D). Furthermore, the motorneuron populations in the Gilbert Randomized networks always settled into periodic activity patterns. However, unlike the Feed and Full Networks, coordinated and synchronized contralateral alternating firing patterns of motorneurons were decimated. This empirically emphasizes the putative intentional design of the underlying wiring of the *C. elegans* connectome toward achieving an important behavioral function. The structural connectivity of the connectome is not random, but has likely evolved in a purposeful way to subserve specific purposes.

To understand how a single pulse stimulation of the ASEL neuron resulted in the firing patterns of the VB and DB classes in Figure 2, we decomposed the network activity into TSeqs (defined in section 4.7). A TSeq represents the causal nodal interactions between a set of start nodes and a set of end nodes. Each TSeq is a walk, of an individual signal, on the graph from start node to end node. TSeqs capture the signaling pathways of the network, as such, they chart the course of a stimulus or any signal through the connectome. Only a subset of all possible walks on a graph are realizable at any given time. Signal walks are constrained by network parameters, such as, edge delays, node refractory periods, and concurrent network activity. We focused on the TSeqs which start at the ASEL neuron, and either terminate or traverse motorneurons from the VB or DB classes. The set of TSeqs contain the relevant neuronal signaling paths (NSPs) which we use to compare dynamics between the Feed and Full networks.

**Figure 3:**
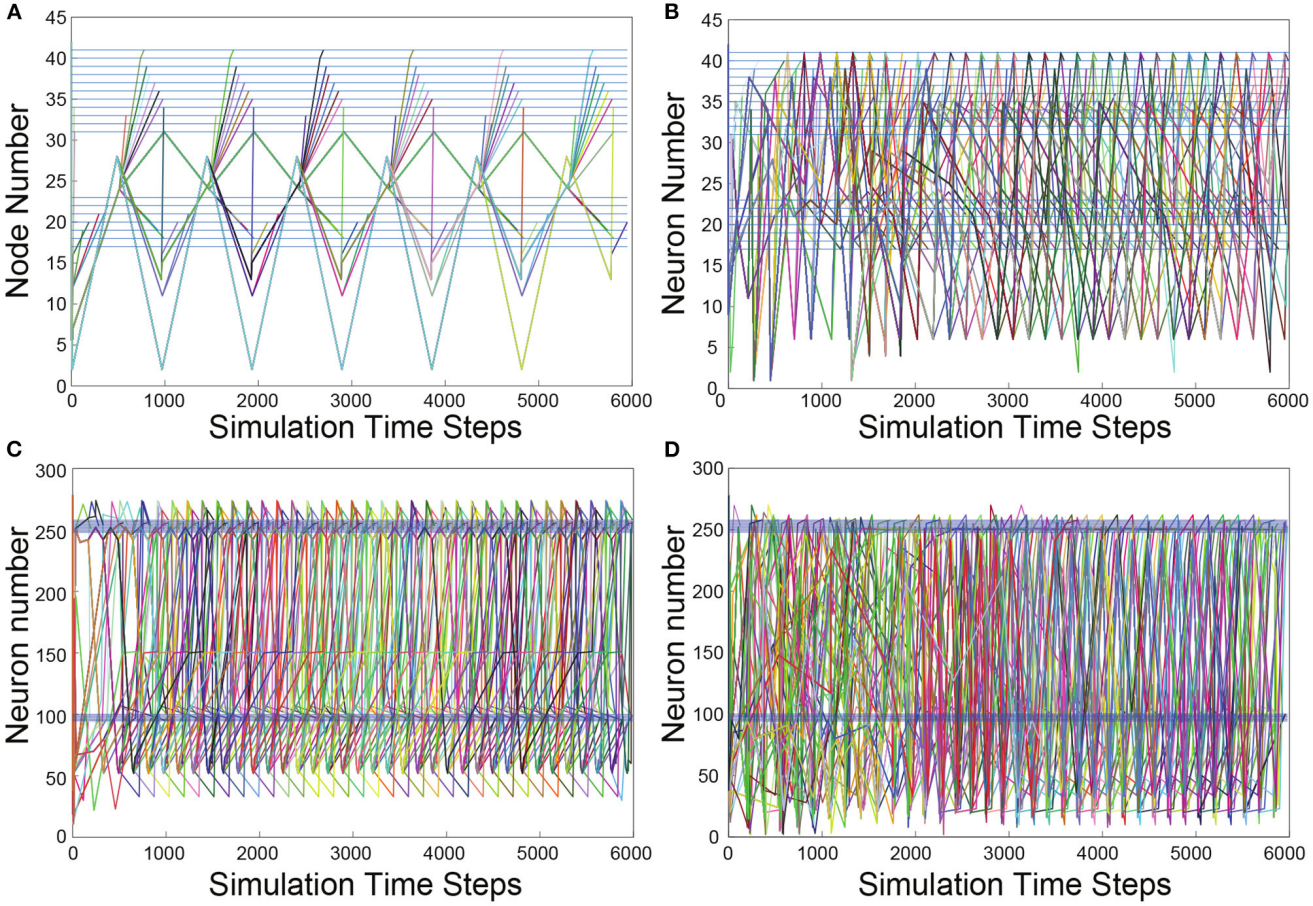
TSeq plots. All TSeqs from ASEL to the VB and DB classes. **(A)** TSeq plot of Feed network. The activation patterns of neurons in the VB and DB classes are synchronized and staggered eventually giving rise to periodic sequences of node activity. **(B)** TSeqs of the Gilbert Randomized Feed network. Synchronized and staggered activations from the VB and DB class are absent. Transient period of network activity is longer than in **(A)**. The TSeqs eventually enter a periodic sequences of node activity. There were significantly more TSeqs resulting from the randomized network in comparison to the TSeqs from the Feed network as in **(A)**. **(C)** The number of TSeqs resulting for stimulating ASEL in the Full network were significantly more than those from the Feed network. Furthermore, the number of motorneurons were activated at a faster rate than they were in the Feed network. This indicates that there are many other signaling pathways or more active signaling pathways from ASEL to the VB and DB classes of motorneurons which are either not captured or not as quickly activated by the Feed network. Qualitatively, the TSeqs of the Full network enter periodic activity within the first 1,000 simulation time steps. **(D)** TSeqs from Gilbert Randomized Full network. The TSeq displayed a significantly longer transient period before the network activity entered a periodic regime. The transient period lasted approximately for the first 2,000 steps of network activity.

To visualize the evolution of TSeqs over time, we created a TSeq plot (defined in section 4.8). In Figure 3, we show the TSeqs ascertained from the different networks we considered. The TSeq plot is similar to a raster plot, where the abscissa is time, and the ordinate is node number. Each curve in the TSeq plots traces the causal walk of a signal on the structural connectome leading to the activation of a neuron of interest. The locations of the motorneurons (end nodes) on the TSeq plot are indicated by the dark blue horizontal lines. All the curves that are active in a time interval trace the concurrent neuronal signaling pathways, which lead to subsequent activations neurons from the classes of interest. To reduce the visual clutter in the TSeq plot we do not explicitly mark the nodes on the walk. The activated nodes are generally located at notches along the curve.

As anticipated, based upon observation of the histogram of node activity in the randomized network (Figures 2B,D), the TSeq plots of the randomized networks show a complete breakdown of NSPs (Figures 3B,D) relative to TSeqs from the Full and Feed networks (Figures 3A,C). Another notable qualitative difference between the TSeqs of the edge randomized and original networks is that the TSeqs of the Full and Feed networks enter a periodic regime of activity significantly sooner. Additionally, the TSeqs in the randomized network displayed convoluted neuronal signaling pathways relative to the orderliness of signaling pathways of the non-randomized networks. Although the histograms of node activations showed a similar trend in class-wise VB and DB motorneuron activations (Figures 2A,C), the TSeq plots show that the NSPs in the Full Network are markedly different (Figures 3A,C). We were motivated by this observation to develop a quantitative approach to compare TSeqs.

### 2.3. Quantitative Comparison of Temporal Sequences

To quantitatively compare TSeqs we developed the Temporal Sequence-Similarity Measure (TSeq-SM). Before presenting the results, first introduce the TSeq-SM algorithm, next we describe a set of networks for which we computed TSeqs, and finally, we discuss the TSeq-SM results.

#### 2.3.1. Similarity Measure Definition

The TSeq-SM is used to quantitatively determine the similarity between any two sets of TSeqs. Sets of TSeqs can be derived from the same network or from different networks. Of the two sets of TSeqs being compared by the TSeq-SM, one of the sets must be a user defined reference set. The TSeq-SM is calculated relative to the length of each TSeq from the reference set. We count the number of TSeqs meeting some matching criteria α, where α is the percent of the length of one sequence from the reference set matching a sequence from the other set. The TSeq-SM is similar to graph edit distances (Sankoff and Kruskal, 1983), but is applied to an ensemble of walks.

Let's say we wish to calculate TSeq-SM between networks X and Y, where X is the reference. The inputs to the algorithms are (*X, Y*, α). The output of this algorithms is the TSeq-SM value. The sets *X* and *Y* contain the TSeqs from networks X and Y respectively, and α is a scalar whose value is between [0, 1]. Let the *i*^*th*^ TSeq of *X* be denoted *x*_*i*_, and the *j*^*th*^ TSeq of *Y* be denoted *y*_*j*_, both *x*_*i*_ and *y*_*j*_ contain some number of node labels, each sequence of node labels representing a sequence of causal activations. Each comparison of the TSeq is relative to a threshold α which is a percentage of total elements in a particular *x*_*i*_ which in-order match the sequence *y*_*j*_ (getTSSimilarityMeasure is defined in Algorithm 1). For each *x* ∈ *X* we evaluate (1) for every *y* ∈*Y*:

(1) $$\begin{array}{r} getTSSimilarityMeasure\left(x_{i},y_{j}\right)\geq \alpha \end{array}$$

The output of (1) is either *True* or *False*. When (1) is *True*, a counter, *c*_α_, is incremented, and the next TSeq from *X*, *x*_*i*+1_ is checked against all *y* ∈ *Y*. The final value of the counter, *c*_α_, is number of TSeqs from X which met the criteria α relative to the TSeqs from Y. For example, if 4 out of 5 node activations in some *x*_*i*_ found an in-order match to some *y*_*j*_, and 4 out of 5 was the best match with respect to all *y* ∈ *Y*, then we say *x*_*i*_ meets the α = 0.8 criteria, and the counter *c*_0.8_ is incremented by 1. In Algorithm 1, we show the procedure to calculate TSeq-SM.

#### 2.3.2. Description of Networks Considered for Similarity Measure

We computed the TSeq-SM between 9 networks, each with varying degrees of similarity to the Full network. All networks were derived from the Full network. Here we briefly describe some of them.

As mentioned in section 2.2, the Feed network is a subset of the Full network. In addition to the previously described Feed and Full Gilbert Randomized networks, we derived two more random networks using an Edge-Swap randomization scheme (described in section 4.4). The two edge randomization procedures resulted in varying degrees of edge reorganization, while maintaining the number of nodes and node locations.

Of the two randomization procedures, the Edge-Swap randomized network is a less drastic network reorganization because the network generation scheme prescribes that each existing edge swap their terminal node with some probability. This procedure is repeated for some number of iterations. Therefore, not all edges need actually be different from the derived network. In addition, the Edge-Swap Random network preserves network parameters, such as, out-degree and network connectedness. The number of iterations determines the structural deviation from the original network. In contrast, the only constraint for the Gilbert based randomization is to maintain approximately the same number of edges between the original and the randomized networks. Therefore, the Gilbert based edge randomization (Gilbert, 1959) is a more drastic form of network reorganization.

Finally, we constructed the Embedded Random Feed networks (detailed description in section 4.6). These network preserves the edge connections of the Full network outside of the Feed subnetwork. The subnetwork within the Full network uses the same randomized edge connection profile as each of the standalone randomized Feed networks. Two Embedded Random Feed networks are generated, one based on the Gilbert Random Feed network and the other based on the Edge-Swap Random Feed network.

#### 2.3.3. Similarity Measure Results

The results of the TSeq-SM are presented in Table 1. First, We will describe the organization of the table, next the results themselves. The reference networks for each TSeq-SM are the antecedents in each of the column headings in the table. The top-half of Table 1 contains the TSeq-SM results of the Gilbert based randomization, and the bottom-half of the table contains the TSeq-SM results of the edge-swap based randomization. Stimulus was applied at the ASEL sensory neuron in all the networks, and the TSeqs of interest were all those which traversed the VB and DB sets of motorneurons.

**Table 1:** Table of similarity measures.

	**Gilbert rand based network**				
**Percent match**	**Feed (124) vs. Feed rand (598)**	**Feed (124) vs. Full (697)**	**Feed (124) vs. Embedded rand feed (649)**	**Full (697) vs. Embedded rand feed (649)**	**Full (697) vs. Full rand (665)**
0.00%	124	124	124	697	697
25.00%	9	78	9	576	10
50.00%	0	33	0	36	1
75.00%	0	13	0	3	0
100.00%	0	6	0	1	0
	Edge-Swapped network				
Percent match	Feed (124) vs. Feed rand (130)	Feed (124) vs. Full (697)	Feed (124) vs. Embedded rand feed (674)	Full (697) vs. Embedded rand feed (674)	Full (697) vs. Full rand (643)
0.00%	124	124	124	697	697
25.00%	50	78	9	688	16
50.00%	6	33	0	614	2
75.00%	3	13	0	422	0
100.00%	1	6	0	0	0

*The columns contain the pairs of networks compared. The rows in the leftmost column of the table represent the percent of a TSeq (the matching criteria α in section 2.3.1) which matched any of the TSeqs from the reference network. The heading for each column are organized as reference network vs. target network. The cells in the table represent the number of TSeqs of the reference network which found a match TSeq from the target network meeting the corresponding row's matching criteria*.

Most generally, as the α-criteria increased, the number of TSeqs meeting the α-criteria decreased. This is an expected result because deviation from the original network structure causes deviation in TSeqs, given that the rest of the signaling parameters are constant. Therefore, fewer TSeqs from more structurally different networks will meet higher α values.

The TSeq-SM between the reference Feed network and the Full network was greater than the TSeq-SM between each of them and their edge randomized counterparts. This is because the Feed network was more directly derived from the Full network. Next, comparing the TSeq-SM between the two forms of network randomization the TSeq-SM values of the reference Full/Feed networks and their Edge-Swap Randomized networks were higher than the TSeq-SM values of the Full/Feed networks and their Gilbert Randomized counterparts.

Surprisingly, although the Feed network is a subset of the Full network, the Feed network in isolation only completely preserved 6 out of 124 TSeqs, i.e., α = 1 that is a 100% match, the remaining 118 TSeqs followed different neuronal pathways. Ideally, the dynamics of the Feed network should be a subset of the dynamics of the Full network. A natural question which arises is: Can we build a better subnetwork? As a starting point, contained in the 697 TSeqs is the necessary set of nodes required to construct a subnetwork. The sufficient set of nodes are those additional nodes involved in the neuronal signaling pathways of the concurrent network activity which ensures the existence of the 697 patterns. Determining the sufficient set of nodes is out of the scope of this paper because that requires iterative simulation and TSeq-SM measurements.

Given the relatively poor TSeq-SM between the Feed and Full networks, we sought to quantify the neuronal signal paths in the Full network traversing nodes outside the Feed network which link ASEL to the VB and DB classes of neurons. There are possible several approaches to generate an intermediate network which bridges the dynamical regime between the isolated Feed network and the Full network. One approach is to remove all the nodes and edges of the Feed network from the Full network, except those nodes associated with ASEL, VB, and DB. If we were to remove all the nodes of the Feed network from the Full network certainly we would find TSeqs which lie completely outside the Feed network. But removing all the nodes and edges associated with the Feed network from the Full network can drastically affect the overall dynamics of the network due to the absence concurrent dynamics. To workaround this issue, we used the Embedded Random Feed network.

Relative to the TSeq-SM values resulting from the comparison of the isolated Feed network (vs. Full network), we observed higher TSeq-SM values while comparing the reference Full network and the Embedded Random Feed networks (Table 1, 5th column). Although we found no TSeqs met the α = 1% criteria, across α values the Embedded Random Feed networks better preserved TSeqs than the reference isolated Feed network relative to the Full network.

Although these results paint a cautionary tale for the analysis of isolated subnetworks. Our quantitative approach of comparing network dynamics provides an indicator of a subnetwork's efficacy. Further analysis of TSeqs provides an avenue for generating subnetworks, as well as preserving specific network interaction patterns.

### 2.4. Decomposing Temporal Sequences Into Basis Sequences

We decomposed the complex patterns of network activity into a basis set of TSeqs (basis set construction is described in section 4.9). In brief, a basis set of TSeqs is a set of composed of TSeqs which arise from signals performing one-time walks and repeating walks on the graph. A repeating walk exist by virtue of signals traversing cycles of the graph. Each of the TSeqs from the set of repeated TSeqs contain sub-sequences which repeat some number of times. We can recompose all TSeqs from the set of One-Time walks and Repeating walks (given the repeating sub-sequences and their repetitions).

**Figure 4:**
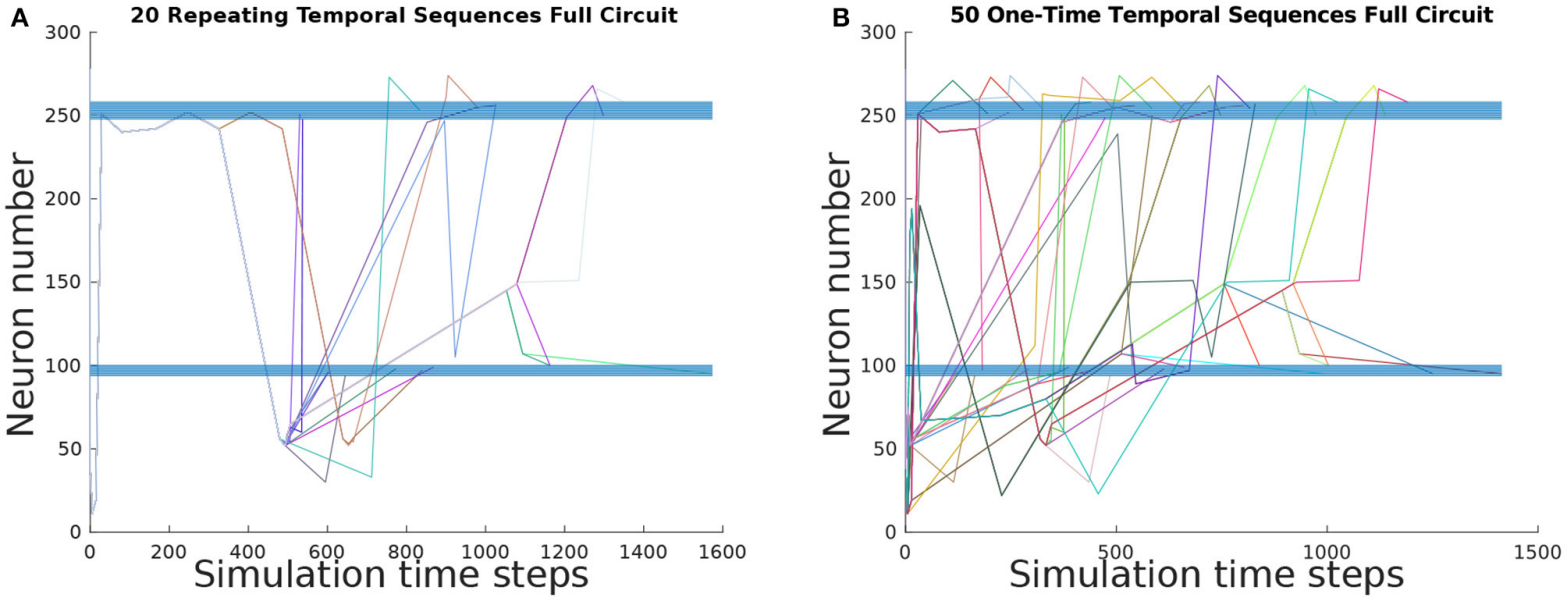
TSeq plots of the basis set of TSeqs from the Full network. The basis set contains One-Time TSeqs and Repeating TSeqs. One-time TSeqs contain TSeqs which represent transient node activity on the network, and the Repeating TSeqs contain TSeqs which represent steady-state or repeating node activity on the network. **(A)** TSeq plot of Repeating TSeqs. These TSeqs contain sub-sequences that when repeated some number of times, that Repeating TSeq is an exact match for another TSeq captured over the course of the network dynamics. **(B)** TSeq plot of One-Time TSeqs. These TSeqs are unique neuronal signaling paths present in the dynamics.

To ascertain the basis set of TSeqs, we categorized each TSeq into One-Time Temporal Sequences, and Repeating Temporal Sequences. Of the 697 observed TSeqs from the Full network, the basis set contained 50 One-Time TSeqs, and 20 Repeating TSeqs. In Figures 4A,B we show the TSeq plot of One-Time and Repeating TSeqs respectively. While the entirety of the One-Time Sequences are displayed in the figure, we only show the Repeating TSeq with a minimum of their repeating sub-sequences.

Each of the 50 One-Time TSeqs are unique because they did not match any of remaining 696 TSeqs (not counting the TSeq being compared). Since there were 50 unique TSeqs, out of 697 total TSeqs, the 20 Repeating TSeqs compose the remaining 647 observed TSeqs through some sub-sequence repetition. Together the One-Time and Repeating TSeq form the minimal description of the dynamics and can be used as for further analysis and network construction.

### 2.5. Interacting Motorneuron Classes

Forward and backward movement of *C. elegans* results from the synchronized activations of banks of motorneurons (Haspel et al., 2010) along its body. The cause of this synchronized activity involves the interaction of several classes of neurons (Chalfie et al., 1985; Zhen and Samuel, 2015). We analyzed TSeqs to identify the interaction between the various classes of mid-body motorneurons. We focused on TSeqs which started at the ASEL neuron, and traversed the mid-body motorneurons from the DA, DD, DB, VA, VD, and VB classes.

**Figure 5:**
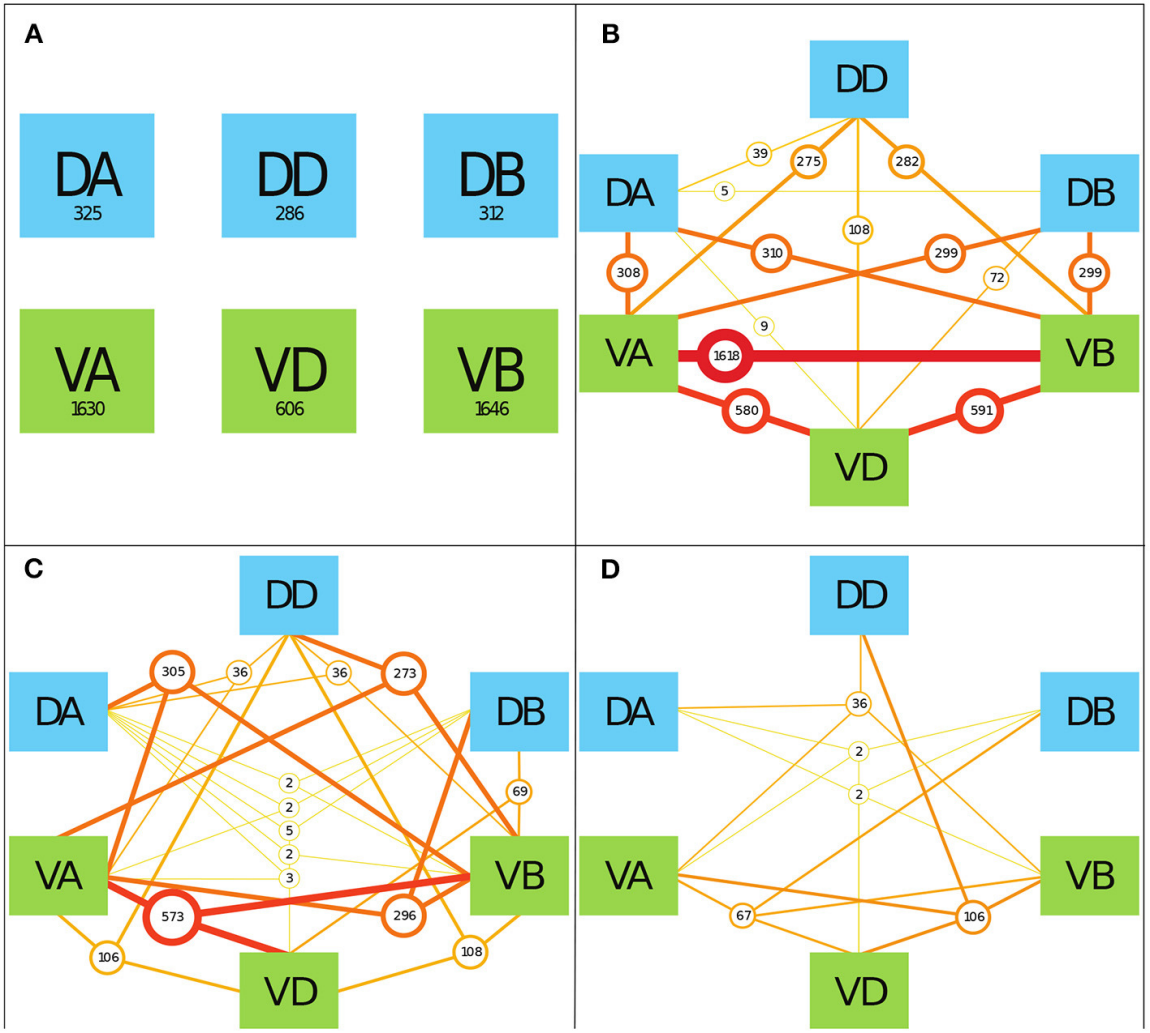
The interaction of various mid-body motorneuronal classes implicated in movement response upon stimulating ASEL in the context of the Full network. We expand the motorneuron classes under consideration to include the DA, DD, DB, VA, VD, and VB classes. We color code the dorsal side classes in blue and the ventral size classes in green. To elucidate the classes implicated in individual TSeqs, as a result of ASEL stimulation, we counted the number of TSeqs which contained at least 1, 2, 3, and 4 classes out of the 6 total classes under consideration. We only show 4 panels because there were no TSeqs which contained 5 or more classes in them. We do not consider the order of present classes because of the much bigger combinatorial space to visualize. **(A)** The values in each of boxes indicate the number of TSeqs with the specific neuronal class present within the TSeq. For example, looking at the box labeled DA, at least one of the neurons in the DA class was present in 325 TSeqs. The ventral classes were over-represented in TSeqs with single classes present. **(B)** Here we considered the presence of any two classes of motorneurons in individual TSeqs. The number of sequences are given by the circles. The most prevalent two classes present were neurons from the VA-VB classes. Only the DB-DD class interaction was not observed in any of the TSeqs captured. **(C)** Here, we considered three class interactions. Here we observe significantly fewer TSeqs than in **(B)**. **(D)** There are significantly fewer TSeqs meeting the four present classes criteria.

To determine the prevalence of various neuronal classes in neuronal signaling paths, we counted the number of TSeqs which contained multiple motorneuron classes. As a starting point, in Figure 5A, we show the number of TSeqs which contain neurons from individual classes. All of the classes implicated in movement were present in at least some of the TSeqs (Figure 5A). Interestingly, the ventral side neuronal classes had significantly more TSeqs traverse their neurons than the dorsal side neuronal classes.

Although the order of classes along the signaling pathways are relevant, they were not considered in this work because of the size of the permutation space. In Figures 5B–D, we show the results for the number of TSeqs with 2, 3, and 4 neuronal classes present. Generally, as the number of classes present in a TSeq increased, the number of TSeqs traversing those classes decreased. Interestingly, individual TSeqs contained at most 4 interacting neuronal classes out of the possible 6 neuronal classes. As such, we find that signaling dynamics on the structural connectome limited the neuronal signaling paths to 4 classes of neurons in the motor circuit and no more.

## 3. Concluding Remarks

As a first attempt to study the affects of a geometric embedding on the dynamics supported by the *C. elegans* connectome, we intentionally focused on the simplest node model applicable within the used signaling framework (Silva, 2019). Although our results indicate that there is value in our approach, laboratory experiments have established that many *C. elegans* neurons may not communicate using discrete signaling dynamics, but rather through graded isopotential dynamics. This is a source of difference between the node models and signaling framework we used, and biophysical models used in other works. None the less, *C. elegans* neurons are physically separated in space and their membrane morphology constrains their neuronal signal propagation velocity, thereby, inducing edge signal delays. A future line of inquiry should be the study of the affects of a geometric embedding on the dynome using more sophisticated node models which bridge the gap between models used here and biophysical models.

Several works have observed that *C. elegans* displays low-dimensional neural activity (Kato et al., 2015; Linderman et al., 2019; Kunert et al., 2014). Similar low-dimensional behavior of node activity would not be expected between the time series of the Full network's motorneuron activity, and time series of the isopotential neuron's voltage dynamics. But through the decomposition of TSeqs into Basis Sequences, we ascertained a low-dimensional representation of the dynamics in the sense of TSeqs. As noted in the results, only 20 Repeating TSeqs gave rise to the observed 647 TSeqs and from the TSeq plots we can see that many of those sequences share significant sub-sequences. As such, we qualitatively observed that the signaling on the geometric network also displays low-dimensionality.

Researchers have not resolved the exact mechanisms of oscillatory dynamics on arises from the *C. elegans* connectome (Gjorgjieva et al., 2014) to sustain locomotion, but there are several hypothesis (Wen et al., 2018; Kunert-Graf et al., 2017; Olivares et al., 2018; Wen et al., 2012) that not only involve the connectome, but also proprioceptive coupling of cells. Although we observed rhythmic behavior of the VB and DB populations of motorneuorns, one must be careful in overgeneralizing from the observations. Since we model the network as a geometric graph, all cycles in the graph are candidate oscillatory circuits. Additionally, we did not introduce any mechanisms for signal degradation, that is how a single impulse stimulus at ASEL resulted in oscillatory dynamics through the realized cycles. Therefore, within the context of our model, a cycle's realization over the course of the dynamics is constrained by signaling parameters and concurrent network activity. These assumptions make any detailed hypothesis of specific signaling paths out-of-scope. Using the idea of the optimal refraction ratio from (Silva, 2019), the range of signaling parameters which induce oscillatory dynamics can be further studied. Future work which uses more granular signaling parameters and/or more sophisticated node models can be in a position to propose oscillatory circuits based on the simulated dynamics on a geometric network.

Several works have focused on detailed subnetwork and whole worm analysis (Kato et al., 2015; Brennan and Proekt, 2019; Kaplan et al., 2018; Wen et al., 2018; Olivares et al., 2018; Kunert et al., 2017). Along the lines of other work (Liu H. et al., 2018), a comprehensive characterization of the dynamics on the *C. elegans* connectome using our signaling framework and methods is needed. Furthermore, the stability and sensitivity of network dynamics to variations in initial conditions, small perturbations, and various stimuli are all interesting research directions, some of these have been pursued in the non-geometric setting (Kunert-Graf et al., 2017).

TSeq based network analysis can be generalized to other scientific disciplines where a network abstraction is possible and causal signaling dynamics can be discerned. In addition to constructing TSeqs, more complex mathematical structures can be built to gain better insights into the structure of network dynamics. TSeqs can easily be extended into graphs and other topological structures (Milo et al., 2002; Alon, 2007; Wong et al., 2012; Curto, 2016). Each of these abstractions provide insights at various temporal and spatial scales.

**Algorithm 1:** Similarity Measure Algorithm

1: **procedure** getSimilarityMeasure(*X, Y*, α)
2: **for** *x*_*i*_ ← 1, |*X*| **do**
3: *maxPercentMatch* ← 0
4: *percentMatch* ← 0
5: **for** *y*_*j*_ ← 1, |*Y*| **do**
6: *percentMatch* ← *getTSSimilarityMeasure*(*x*_*i*_, *y*_*j*_)
7: **if** *maxPercentMatch* < *percentMatch* **then**
8: *maxPercentMatch* ← *percentMatch*
9: **if** *maxPercentMatch* ≥ α **then**
10: *count* ← *count* + 1
11: *thresholdMet* ← 1
12: **break**
13: end **if**
14: end **if**
15: end **for**
16: end **for**
17: return *count*
18: end **procedure**

19: procedure getTSSimilarityMeasure(*x*_*i*_, *y*_*j*_)
20: Sequences *x*_*i*_ and *y*_*j*_
21: ⊳ Compares the similarity of Temporal
22: *cardx* ← |*x*_*i*_| ⊳ Store the number of elements in *x*_*i*_
23: *cardy* ← |*y*_*j*_| ⊳ Store the number of elements in *y*_*j*_
24: *lasty* ← 1
25: *count* ← 0
26: **for** *u* ← 1, *cardx* **do** ⊳ We sweep through all the elements of the TSeq *x*_*i*_ looking for in-order matches of elements in *x*_*i*_
27: **for** *w* ← *lasty, cardy* **do**
28: **if** *x*_*i*_[*u*] = *y*_*j*_[*w*] **then**
29: *count* ← *count* + 1
30: *lasty* ← *y*_*j*_[*w*]
31: else
32: *continue* ⊳ Go to the next iteraction of *w* for-loop
33: end **if**
34: end **for**
35: end **for**
36: *PercentMatch* = *count*/*cardx*
37: return *PercentMatch*
38: end **procedure**

## 4. Methods

### 4.1. Full Network Construction

Our computational model builds upon the experimentally derived *C. elegans* connectome (White et al., 1986). We call the network model of the entire structural connectome the Full network. All other networks in this work are derived from the Full network. Mathematically, we treat the connectome as a directed geometric graph (Diestel, 2006; Silva, 2019). A graph *G* is a pair *G* = (*V, E*). Where *V* is the set of nodes/neurons, and *E* is the set of edges/axons such that *E* ⊆ *V* × *V*. A geometric graph is a graph whose nodes are embedded in euclidean space. A network is an applied instance of a graph, in our case a biological neuronal network, the *C. elegans* connectome. A directed network representation was used because signals along axons generally travel unidirectionally (Kandel et al., 2013). We used a simplified version of the Geometric Dynamic Perceptron model to describe the dynamics of each node (Silva, 2019).

We constructed the directed adjacency matrix of the Full network using publicly available connectivity information (White et al., 1986; Altun et al., 2002–2017; Kaiser and Hilgetag, 2006; Varshney et al., 2011). Let *A* be the network adjacency matrix, if there is a connection between neuron *i* and neuron *j*, then *A*_*ij*_ = 1, otherwise *A*_*ij*_ = 0. Although neurons communicate with each other through multiple modes, our model only incorporates chemical/axonal connections between neurons. We do not consider electrical connections through gap junctions. Combining the structural connectivity with the location of neuronal cell bodies (Choe et al., 2004) we constructed the distance adjacency matrix. Out of the 302 neurons which make up the hermaphrodite *C. elegans* connectome, only 277 neuron locations are known (Choe et al., 2004). Therefore, our final network consists of 277 nodes. We calculated the edge length using a euclidean distance between somatic bodies.

Although, there is much debate as to whether *C. elegans* neurons use action potential like signaling, for simplicity, we assumed that some quanta of charge is transmitted between nodes through all-or-none stereotyped nodal events (Mellem et al., 2008, 2009; Lockery and Goodman, 2009; Lockery et al., 2009). Once a node initiates a signal, the signal traverses all outgoing edges at a constant conduction velocity. We assumed a conduction velocity of $80\frac{mm}{s}$ which is within the theoretical range for this type of organism (Niebur and Erdo, 1993). We calculated the signaling time delays along edges through the geometrically derived edge lengths and the conduction velocity.

All nodes in our network model are either inhibitory or excitatory. Excitatory nodes propagate excitatory signals through their edges. The response to an incoming excitatory signal is either the activation of the node receiving that signal, or the addition of potential to the receiving node's membrane potential. If the receiving node's membrane potential has exceeds threshold, the node generates signals which propagate along all outgoing edges, immediately the node becomes refractory for the duration of its refractory period. The response to an incoming inhibitory signal is similar except no outgoing signals are generated by the receiving node. Only the GABA expressing neurons were considered inhibitory (Gendrel et al., 2016). All other neurons types were considered excitatory (Pereira et al., 2015), including the unknown neuron types. While a receiving node is refractory, no incoming signals will contribute to its firing. The refractory period of all neurons is set to 4*ms* (Berry and Meister, 1998; Mack et al., 2013).

In our simplified version of the Geometric Dynamic Perceptron node model, the state of node *i* at time *t* be given by *y*_*i*_(*t*) = {0, 1}. When the node is in the active state *y*(*t*) = 1, when it is inactive *y*(*t*) = 0. While node *i* is initially in an inactive state it can instantly transition to the active state; but when the node *i* is in the active state it will become refractory for the time interval of the refractory period. We set the threshold for node activation to be one incoming signal. Therefore, when a signal arrives to the axon hillock of node *i* and it is in the inactive state, instantaneously, an outgoing signal will be generated and transmitted through all its outgoing edges.

We initialize network activity to quiescence. We initiate network activity by stimulating the ASEL neuron with a single pulse stimulus, eliciting outgoing signals based on the network connectivity. The simulation is run for approximately 0.15*s* of network activity, broken up into 6000 discrete time steps.

### 4.2. Feed Network Construction

The Feed network (Xu and Deng, 2013) is a hypothesized network which contains the neurons governing movement response to a feed stimulus. The Feed network is subset of the Full network. The Feed network contains the chemosensory neuron ASEL, interneurons, and motorneurons. We reused the physical parameters from the Full network for the Feed network.

### 4.3. Lattice Network Construction

We constructed the Lattice network to study the affect of edge lengths on dynamics. The Lattice network is derived from the Full network. While we used the adjacency matrix of the Full network in the Lattice network, we modified the signaling parameters of the Lattice network. Specifically, we set all edge delays of the Lattice network to 1*tu* (arbitrary time unit) and we set all node refractory periods to 0.9*tu*. This ratio of refractory period to signaling delay is considered to approach optimal from a local signaling perspective (Silva, 2019).

### 4.4. Edge-Swap Random Network Construction

We constructed a random network called the Edge-Swap Random network using the edge-swapping algorithm from Rubinov and Sporns (2010). The the randomization procedure incrementally modifies the original network's connectivity. We briefly describe the randomization procedure.

Two edges are randomly selected. If the two edges have two distinct source nodes and two distinct destination nodes, then the two destination nodes are swapped, only if the new edges formed by the swap do not already exist. If any of the nodes associated with the randomly selected edges are the same, then two other edges are randomly selected, and the procedure is repeated. When an edge is successfully modified, a network connectedness check is performed to make sure a separate network component was not created during the edge-swapping process. The process is repeated approximately as many times as the number of edges in the network. Each cycle through all the edges is consider an iteration of the algorithm. The original and randomized networks diverge in connectivity as an increasing function of the number of iterations. The code for the edge-swapping algorithm can be found at Rubinov and Sporns (2010).

We ran the algorithm for 10 iterations on both the Full network and the Feed network, to generate their randomized counterparts. This randomization scheme preserves the node degree distribution, the number of edges, and network connectedness. Finally, we computed the distance adjacency matrix from the adjacency matrix of the last iteration of the algorithm.

### 4.5. Gilbert Randomized Network Construction

The Gilbert Randomized network (Gilbert, 1959; Erdos and Renyi, 1960; Prettejohn et al., 2011) is derived from the Feed and Full networks. The Gilbert random networks approximately preserved the total number of edges in the Feed and Full networks, but the edge between nodes is randomly drawn from the set of all possible edges between any of the nodes in the network. We calculated the probability of edge connection as follows: Let *N* be the number of nodes in the Full network, and let *e* be the number of edges in the Full network.

(2) $$\begin{array}{r} p=\frac{e}{N\left(N-1\right)} \end{array}$$

To create this network's adjacency matrix, we created a matrix of random real numbers between 0 and 1. Each value in the matrix is determined by using *p* as the threshold value. If the matrix entry is greater than *p*, then we set the values of the matrix entry to 0, otherwise, the matrix entry is set to 1. This results in an adjacency matrix of 0s and 1s with approximately the same number of edges in the Gilbert Randomized network as in the Full network. Using the spatial location data for the neurons, we calculated a distance adjacency matrix based on the final network connectivity. The signaling parameters of the Gilbert Randomized networks are the same as those of the Full network.

### 4.6. Embedded Random Feed Network Construction

We embedded the (Gilbert or Edge-Swap) randomized Feed network into the Full network to generate the Embedded Random Feed network. We constructed this network by replacing the rows and columns of Full network's distance adjacency matrix with the values from the distance adjacency matrix of the randomized Feed networks. All the signaling parameters of the Full network were reused for the Embedded Random Feed network.

### 4.7. Temporal Sequence Construction

A Temporal Sequence (TSeq) is a specific type of walk on a graph (Diestel, 2006). This walk follows the trajectory of a quanta of signal between nodes along edges on a graph. In our network setting, TSeqs describe the trajectories of discrete signals, along edges, connecting nodes. The trajectory consists of a set of nodes traversed by an signal emanating from an initial node activation. Since we were interested in TSeqs which traversing the Feed network, we specified ASEL as the start node, and the VB and DB motorneurons as the end/traversed nodes. The resulting set of TSeqs represent the parallel paths through the graph traversed by signals beginning at the start node and traversing the end nodes over a time interval of interest.

For example, let us say we have a graph *G* = (*V, E*), a pair, formed by a set of nodes *V*, and edges *E*. The elements of *G* consist of *v* ∈ *V* and *e* ∈ *V* × *V*. At time *t* = 0 lets say that node *v*_0_, some element in *V*, activates. Assume that *v*_0_ has an outgoing edge to *v*_1_, once the signal from *v*_0_ reaches *v*_1_, *v*_1_ activates, which in turn generates an signal. Lets say that this process continues until at some time *t* = *t*_*f*_ when node *v*_*t*_*f*__ activates. The sequence of nodes *X*_*t*_*f*__ = (*v*_0_, *v*_1_ ⋯ *v*_*t*_*f*__) is the TSeqs starting at *v*_0_(start node) traversing *v*_*t*_*f*__(end node).

### 4.8. Temporal Sequence Plot Description

We construct a plot similar to the spike raster plot to visualize the evolution of Temporal Sequences. We call this new plot a Temporal Sequence plot (TSeq plot). It has time steps on the x-axis, and node number on the y-axis. Each curve represents the causal trajectory of node activity, represented by a TSeqs, over time. We separate each TSeq curve by color.

The activation of different end nodes of a network can result from shared signaling pathways. Those TSeqs contain shared sub-sequences, these are represented by overlapping curves on the TSeq plot. For example, in Figure 4A, we observe that all the Repeating TSeqs go through the same set of nodes at stimulus onset, before splitting into various Basis TSeqs.

In order to better visualize the paths through the nodes of interest, we drew blue horizontal lines on the TSeq plots. Each horizontal blue line is at the height of a neuron from to the VB and DB sets of neurons. In the Feed network the DB neurons are located on the y-axis at values between 17 and 23, while the VB neurons are located on the y-axis at values between 31 and 41. In the Full network the DB neurons are located on the y-axis at values between 94 and 100, while VB neurons are located on the y-axis at values between 248 and 258.

### 4.9. Basis Sequence Construction

The Basis Temporal Sequences (Basis TSeqs) are derived from the original set of TSeqs captured over the simulation time interval of interest. Basis TSeqs consist of two sets of sequences, the set of One-Time Temporal Sequences (One-Time TSeqs) and the set of Repeating Temporal Sequences (Repeating TSeqs). First, we describe the intuition behind each of the constituent sets of sequences, then we will define them in more detail. Repeating sub-sequences result from signals traversing cycles on a graph since no node can spontaneously generate signals (discounting external stimuli). The nodes and edges on a closed walk can give rise to repeating network activity, therefore, they are candidate pattern generators of the network. Some TSeqs which result from signal traversals of graph cycles may only differ from one another by the number of repeating sub-sequences. For each TSeq which differ in repeating sub-sequences, we create a compressed TSeq. We compose a set of all the compressed TSeqs and call it the set of Repeating TSeqs. Given the description of the set of Repeating TSeqs, we can readily describe the set of One-Time TSeqs as the remaining TSeqs which are not Repeating TSeqs.

More formally, let *X* be the set of all TSeqs. Let *R* be the set of Repeating TSeqs in *X*, *R* ⊆ *X*, and let *U* be the set of One-Time TSeqs, *U* ⊆ *X*. The sets *R* and *U* are such that *R* ∩ *U* = ∅. That is to say no TSeqs can be a member of both the Repeating TSeqs and the One-Time TSeqs. The members of sets *R* and *S* are described next.

To determine whether some *x*_*i*_ ∈ *X* is a member of *R* we analyze the sub-sequences in *x*_*i*_. The result is that for some *r*_*i*_∈*R* and *r*_*j*_ ∈ *R*, the only difference between *r*_*i*_ and *r*_*j*_ is in the repetition of some sub-sequences. To determine membership of *x*_*i*_ in *R*, we need to construct a dictionary of candidate sub-sequence repetitions. Let us assume that *x*_*i*_ contains sub-sequences which repeat in different parts of its sequence. Let *I* = {*i*_1_, *i*_2_ ⋯ *i*_*n*_} be the set of repeating sub-sequences of *x*_*i*_. Where each *i* ∈ *I* is the sub-sequence but without repeats, that is, each element of *I* is a primitive sequence. To account for the repeats, let *K* = {*k*_1_, *k*_2_ ⋯ *k*_*n*_} be the set containing the number of repetitions of each of the primitive sequences in *I*. Now, any *x* ∈ *X* can be re-written as combination of primitive sequences together with its non-repeating sub-sequences. Concretely, given some TSeq *x*, we compress all its repeating sub-sequences in the form of $i_{j}^{k_{j}}$, where *j* is the *j*^*th*^ sub-sequence in *I*, which takes on *k*_*j*_ repetitions.

Every TSeq, *x*_*i*_ ∈ *X*, which can be derived from changing the number of repetitions of a primitive sequence, to match another sequence, *x*_*j*_ ∈ *X* is considered a Repeating TSeq. Both *x*_*i*_ and *x*_*j*_ are considered elements of *R*. Contained in the set *I* are the candidate pattern generators (Marder and Bucher, 2001; Guertin, 2013) of the network, that is, the sequence of node activations which sustain ongoing network activity. These are precisely the realized cycles of the graph. It is not necessary that the dynamics realizes all the possible cycles of the graph. Since the elements of *R* are attained from observing network dynamics, elements of *R* are not guaranteed to repeat indefinitely, therefore, Repeating TSeqs do not necessarily represent true periodic network activity.

Given the definition of Repeating Sequences, we can define the set *U* of One-Time TSeqs as *U* = {*u*|*u* ∈ *X, u* ∉ *R*}. The difference between *R* and *U* is that for all *x*_*i*_ ∈ *U* there is no other sequence *x*_*j*_ ∈ *X* such that the difference between *x*_*i*_ and *x*_*j*_ is only in some repetition of a sub-sequence in *x*_*i*_. Note, *u*_*i*_ ∈ *U* may contain repeating sub-sequences. Since there are many approaches to ascertain Basis TSeqs, we do not provide an explicit algorithm to extract Basis TSeqs. It is possible to reconstruct all TSeqs in *X* form the Basis TSeqs and knowledge of the number of repetitions of each sub-sequence.

## Data Availability Statement

The raw data supporting the conclusions of this article will be made available by the authors, without undue reservation.

## Author Contributions

VG and GS conceived of the study. VG carried out all the analyses. All authors contributed to the writing and experimental designs.

## Conflict of Interest

The authors declare that the research was conducted in the absence of any commercial or financial relationships that could be construed as a potential conflict of interest.
